# Supplementary material for: Long-term perceptual priors drive confidence bias that favors prior-congruent evidence
Source: PLoS Comput Biol. 2025 Dec 22;21(12):e1013826. doi: 10.1371/journal.pcbi.1013826 (PMC12753060; doi:10.1371/journal.pcbi.1013826)
Supplement: S1 Text — Manipulation check per participant; Psychometric functions fit to motion direction decisions per participant; Non-parametric analysis of confidence bias per participant; Confidence choice results split by stimulus and perceptual decision; Posterior Mean model; Correlation between perceptual bias and confidence bias; Condition-specific Likelihood model; CFC-model parameters; BIC results; Model recovery analysis. (DOCX) [file pcbi.1013826.s001.docx]

**S1 Text – Supporting Information**

**Long-term perceptual priors drive confidence bias that favors prior-congruent evidence**

Marika Constant*^a,b^, Elisa Filevich^a,b,c^, Pascal Mamassian^d^

^a^ Humboldt-Universität zu Berlin, Faculty of Life Sciences, Department of Psychology, Berlin, Germany

^b^ Bernstein Center for Computational Neuroscience Berlin, Berlin, Germany

^c^ Hector Institute for Education Sciences & Psychology, University of Tübingen, Tübingen, Germany

^d^ Laboratoire des Systèmes Perceptifs, Département d’Études Cognitives, École Normale Supérieure, Paris Sciences et Lettres University, CNRS, Paris, France

* marika.constant@gmail.com

1. **Manipulation check per participant**

**
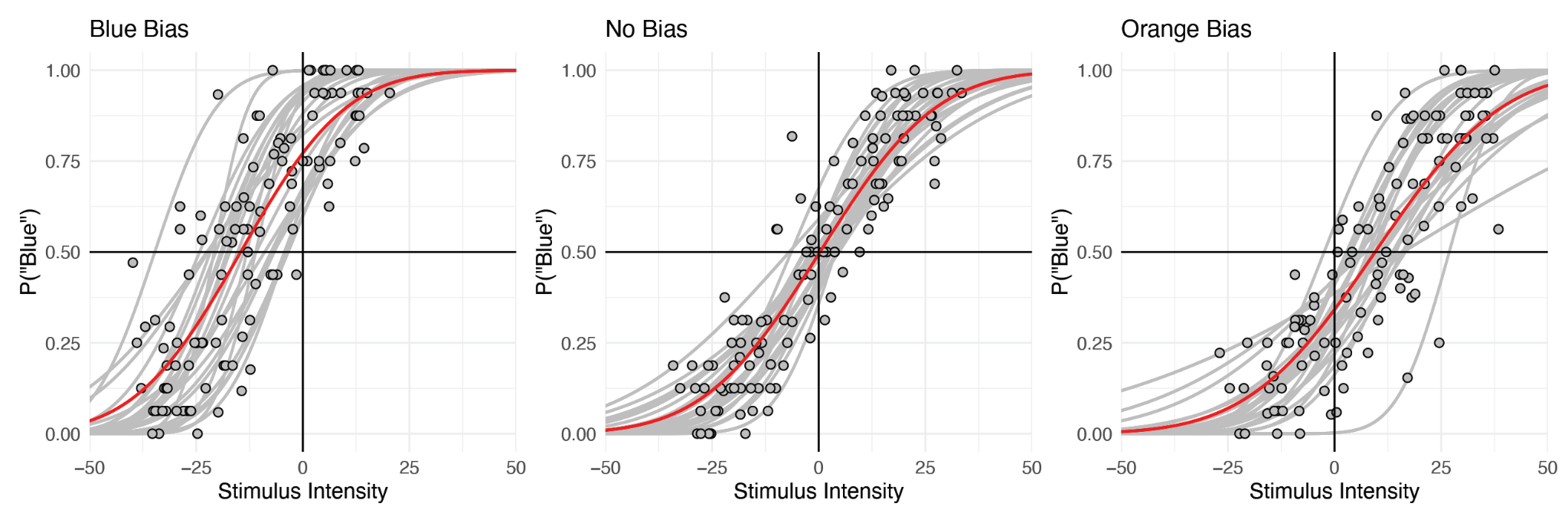
**

**Fig A. Participant-wise manipulation check.** Results from the adaptive staircase (ASA) procedure for each condition across all participants. Each gray point corresponds to the responses for a given stimulus intensity level from one participant. θ values towards the blue region are encoded as positive, and towards the orange region are encoded as negative. Red psychometric functions show the fitted cumulative normal distribution functions to the relationship between θ and the probability of choosing blue from the pooled data. The gray psychometric functions capture the fitted cumulative normal distribution function to data from each individual participant.

1. **Psychometric functions fit to motion direction decisions per participant**


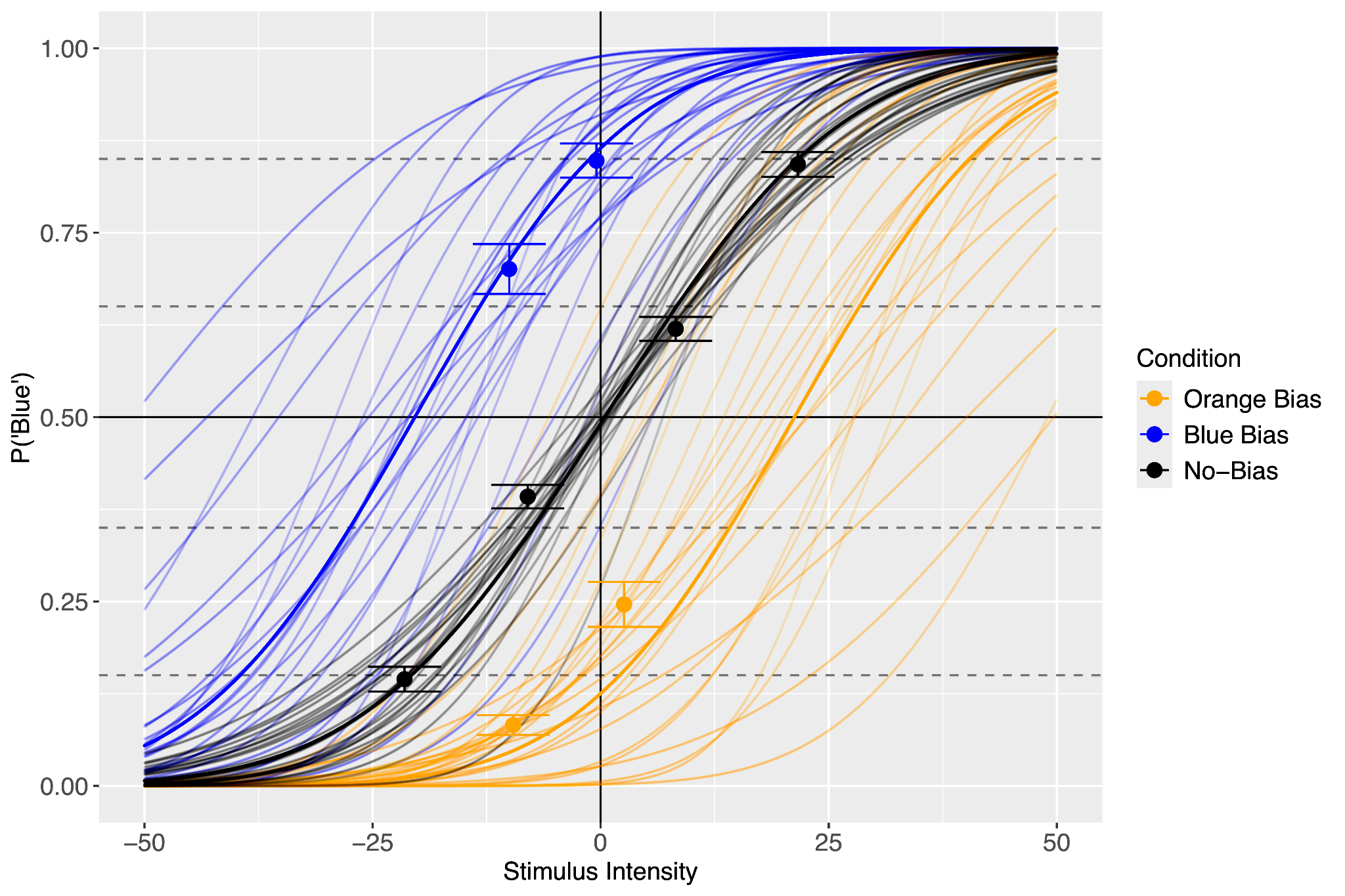


**Fig B. Participant-wise psychometric fits to motion direction decisions.** The psychometric functions were fitted to each individual participant to capture the strength of the bias from the slow-motion prior relative to the No-Bias baseline, and to capture possible differences in sensitivity between conditions. The resulting fits for each participant are shown, with the mean fits across participants shown in the darker lines, matching Fig 2A.

1. **Non-parametric analysis of confidence bias per participant**


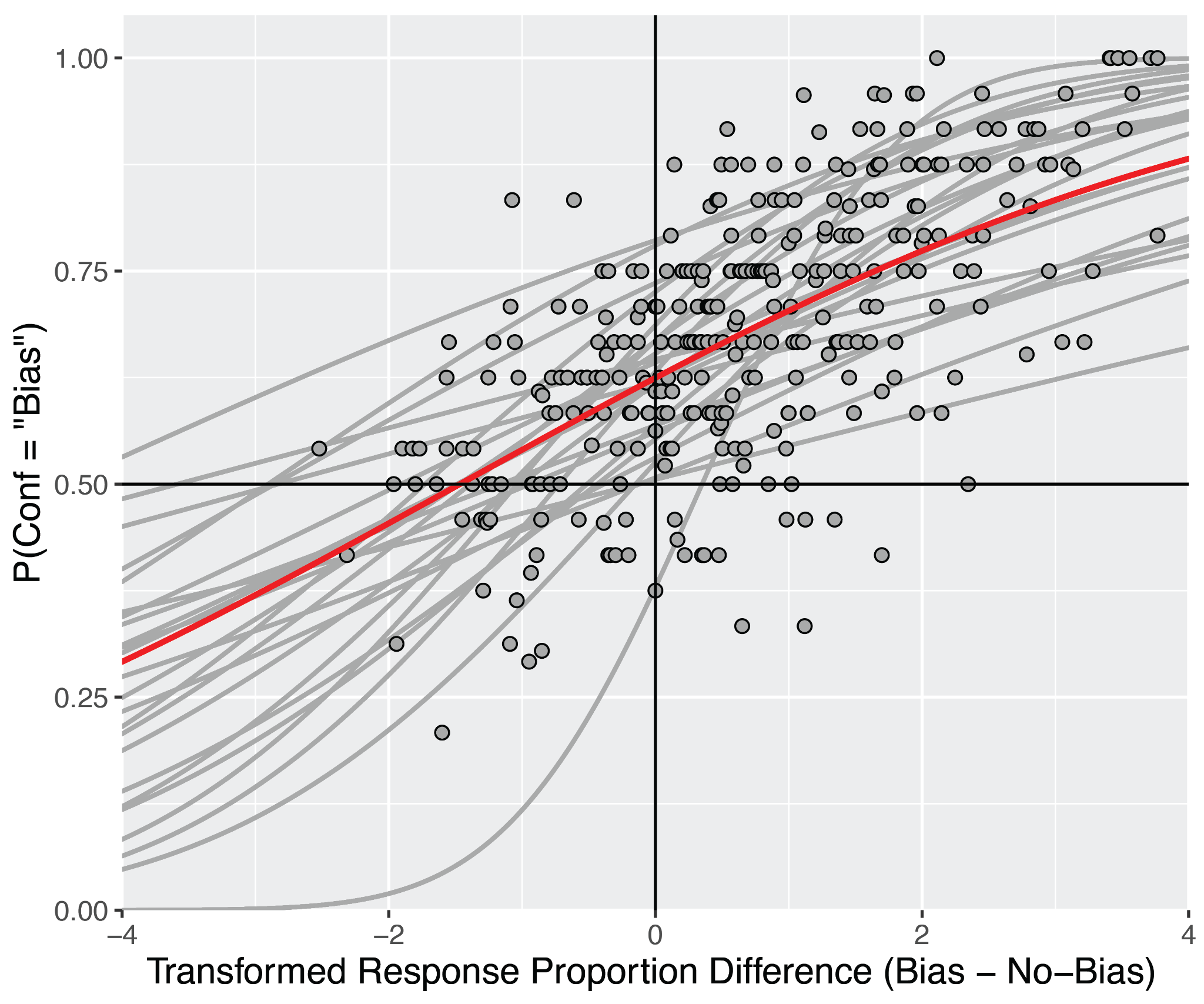


**Fig C. Participant-wise non-parametric analysis of confidence bias.** On the x axis is the difference in the logit transformed rate of choosing the expected color between the Bias and No-Bias condition. Each gray point represents this difference for one pair of stimulus settings with one interval in each condition, from one participant. On the y axis is the probability of choosing the Bias condition interval as the more confident interval. The red psychometric function captures the fit cumulative normal distribution function to the relationship between this response proportion difference and the confidence choice rates from the pooled data, and the gray psychometric functions capture the fit cumulative normal distribution function to each individual participant.

1. **Confidence choice results split by stimulus and perceptual decision**


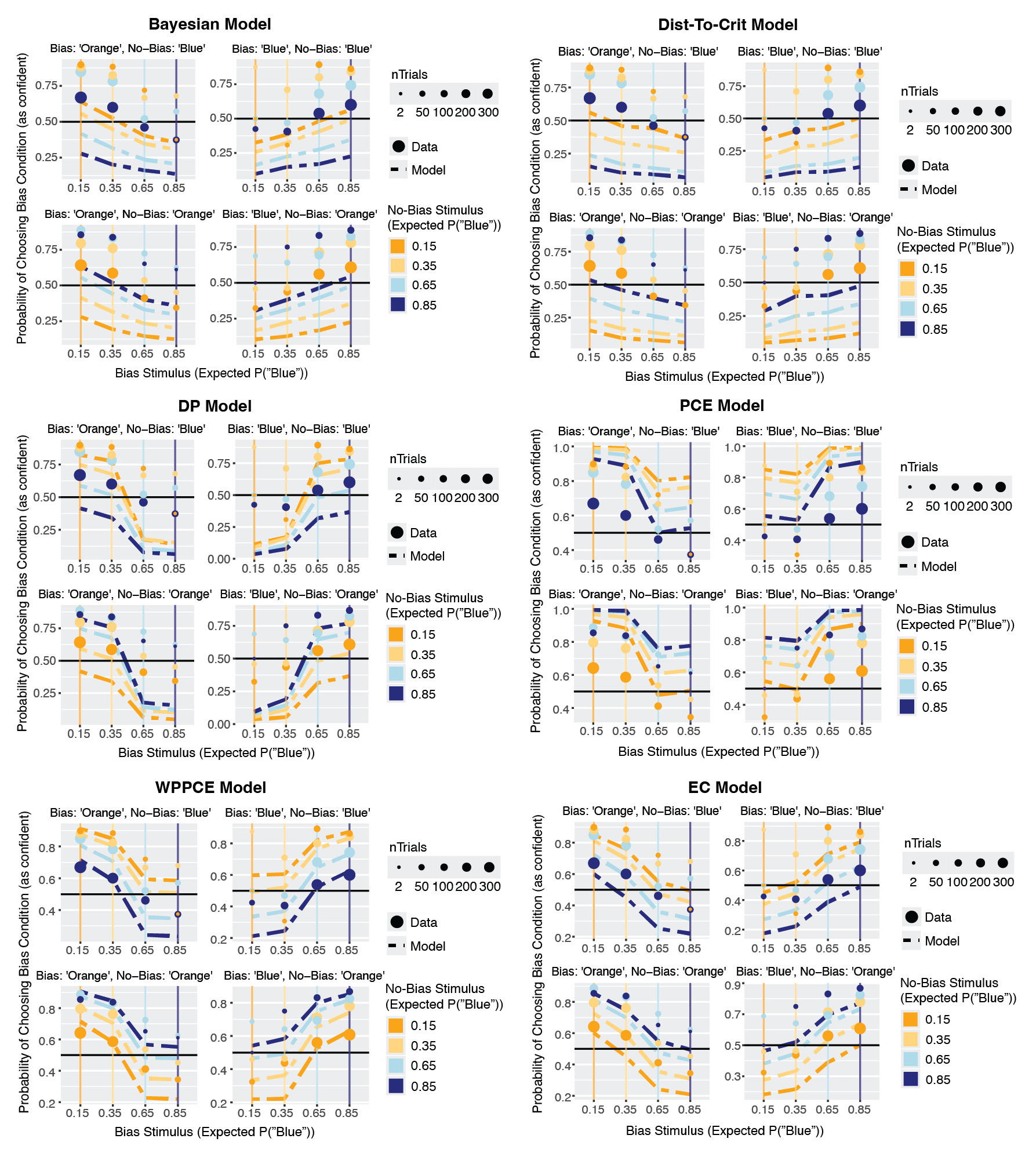


**Fig D. Confidence choice results per stimulus and perceptual decision.** Model predictions against the confidence data for each model. Because confidence refers to an estimate that a perceptual decision is correct, we consider separately each possible combination of orange/blue perceptual decisions across the two intervals (thus 4 panels for all the possible combinations). The x-axis captures the stimulus setting in the Bias condition interval (from targeting most orange decisions on the left to targeting most blue decisions on the right —also indicated by thin colored vertical lines—). The stimulus setting in the No-Bias condition interval is captured by color (from targeting most orange decisions in dark orange to targeting most blue decisions in dark blue). Stimulus settings are defined in units of the expected probability of choosing blue (Expected P(‘Blue’)) from the ASA staircase procedure, or in other words, the targeted perceptual decision rate. For example, an Expected P(‘Blue’) of 0.65 refers to the θ value that was chosen from the staircase to target a blue decision rate of 0.65 in that condition. The y-axis captures the confidence choice rates favoring the Bias condition. Simulated confidence choice rates from each model are shown by the dashed lines and the observed confidence data are shown by circles whose size reflects the number of trials.

1. **Posterior Mean model**

Another possible confidence heuristic is to base confidence off of the distance between the criterion and the mean of the posterior. This is similar to the Dist-To-Crit model, which instead uses the distance between the criterion and a single sample from the posterior. Although computing the posterior mean is more computationally costly, it is still a possibility that is commonly considered. In the Posterior Mean model, high confidence decisions occur when the mean of the posterior is far from the criterion. The confidence forced-choice is then based on which of these confidence values is higher. Like the Dist-To-Crit model, this Posterior Mean model substantially underpredicts confidence choice rates favoring the Bias condition, and performs worse than the Bayesian model, with an AIC = 36855.60.


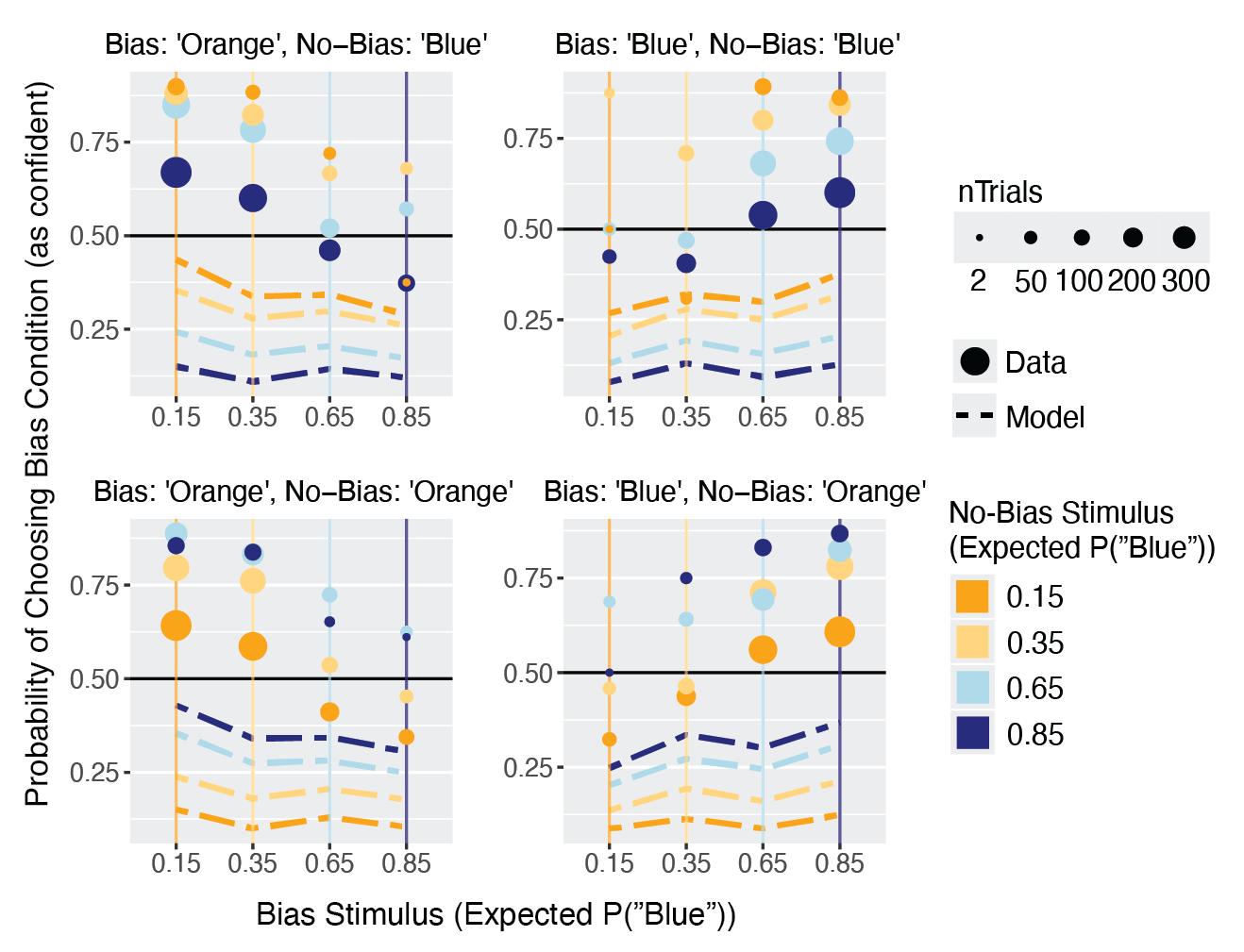


**Fig E. Confidence predictions from Posterior Mean model against data.** Confidence forced-choice predictions simulated from the Posterior Mean model (dashed lines) against the confidence data (points). This model cannot account for the observed confidence bias favoring the Bias condition, and actually predicts an opposite confidence bias favoring the No-Bias condition.

1. **Correlation between perceptual bias and confidence bias**

In order to investigate a possible relationship between the strength of the perceptual prior and the degree of confirmatory confidence bias, we ran a Spearman’s rank correlation analysis. We assessed the correlation across participants between the first-order bias, as quantified by the μ parameter of the fit psychometric functions shown in (Fig B), and the confidence bias, as quantified by the ɑ parameter of the WPPCE model. Note that μ was adjusted to be relative to the No-Bias condition criterion for each participant, to get the degree of perceptual bias *due to the slow-motion prior*, not from other decision biases occurring across conditions. Larger values of μ indicate a stronger first-order effect of the prior and large values of ɑ indicate more use of the PCE rather than the posterior evidence, and hence a stronger confidence bias (relative to first-order decisions). We found a trend towards a positive relationship between the variables, ⍴(24) = 0.40, p=0.055, however this did not reach significance. While we cannot draw conclusions on this basis, it would be interesting to investigate further using an individual differences approach in a larger sample.


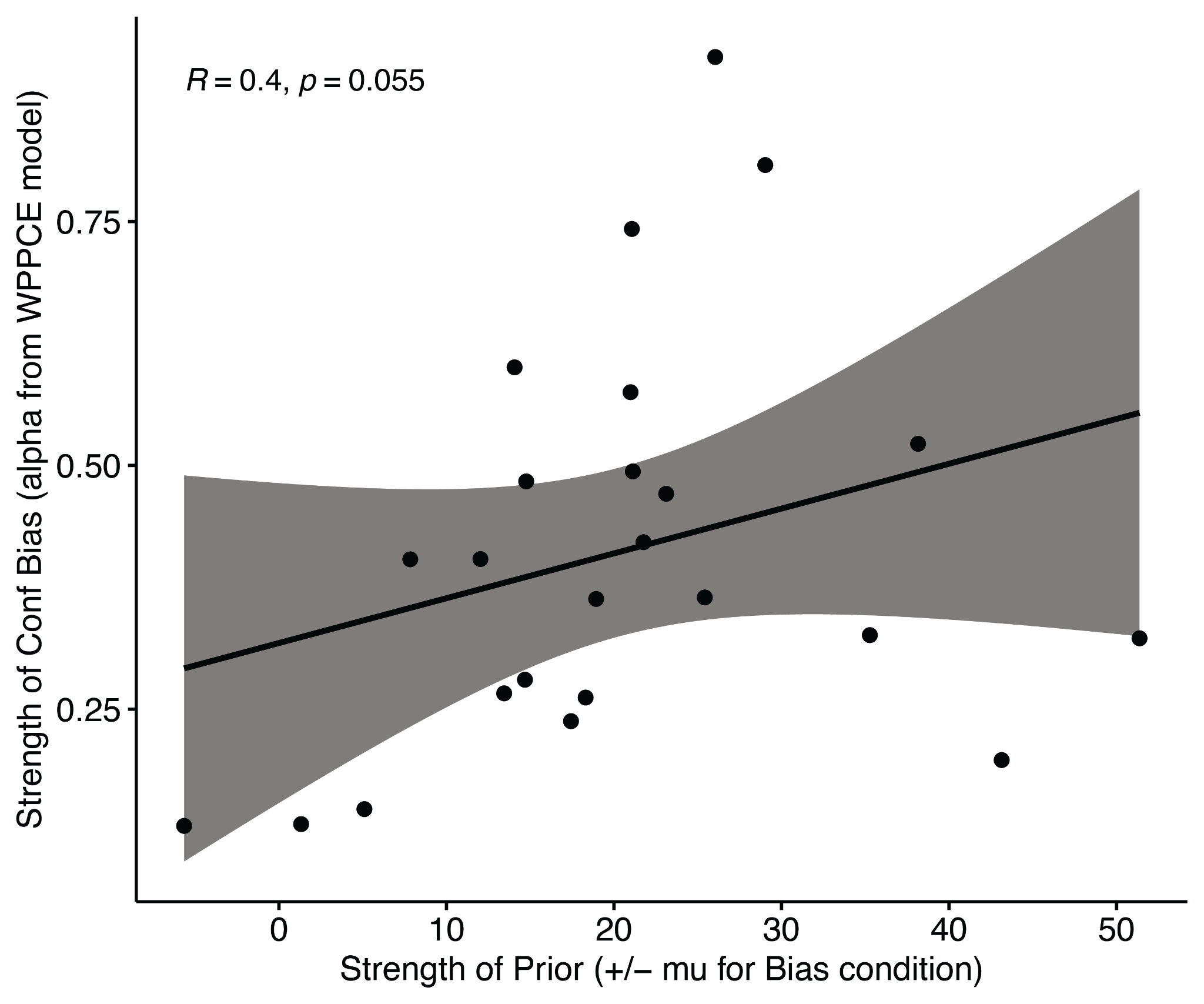


**Fig F. Correlation between strength of the perceptual and confidence bias.** On the x-axis are the μ values from the fit psychometric functions to each participants’ first-order decisions, which capture the degree of criterion shift in the Bias condition compared to the No-Bias condition. On the y-axis are the ɑ parameter values from the WPPCE model fit to each individual participant, which capture the use of the prior-congruent information relative to the posterior evidence and hence reflect the degree of the confirmatory confidence bias. Points reflect individual participants. The solid line represents the Spearman correlation’s regression line and the surrounding shaded area indicates the 95% confidence interval. The Spearman’s rank correlation coefficient and corresponding p-value are displayed in the top-left.

1. **Condition-specific Likelihood model**

To investigate possible differences in the precision of the *likelihood* across the different conditions due to efficient encoding, but without committing to a particular implementation-level model, we fit a version of the Bayesian decision model that allowed for different likelihood noise $\sigma_{L}$ in each condition. The model and fitting procedure were otherwise identical to that of the Bayesian model. The model was fit to the perceptual decisions of each participant, yielding a median $\sigma_{L-Bias}$ = 19.01, a median $\sigma_{L-NoBias}$ = 20.62, and a median $\sigma_{P}$ = 27.96. The slightly lower variance of the Bias condition may point to some form of efficient coding. However, we simulated the confidence patterns expected from this model (Fig G), and it still cannot capture the confidence bias seen relative to the decision level.


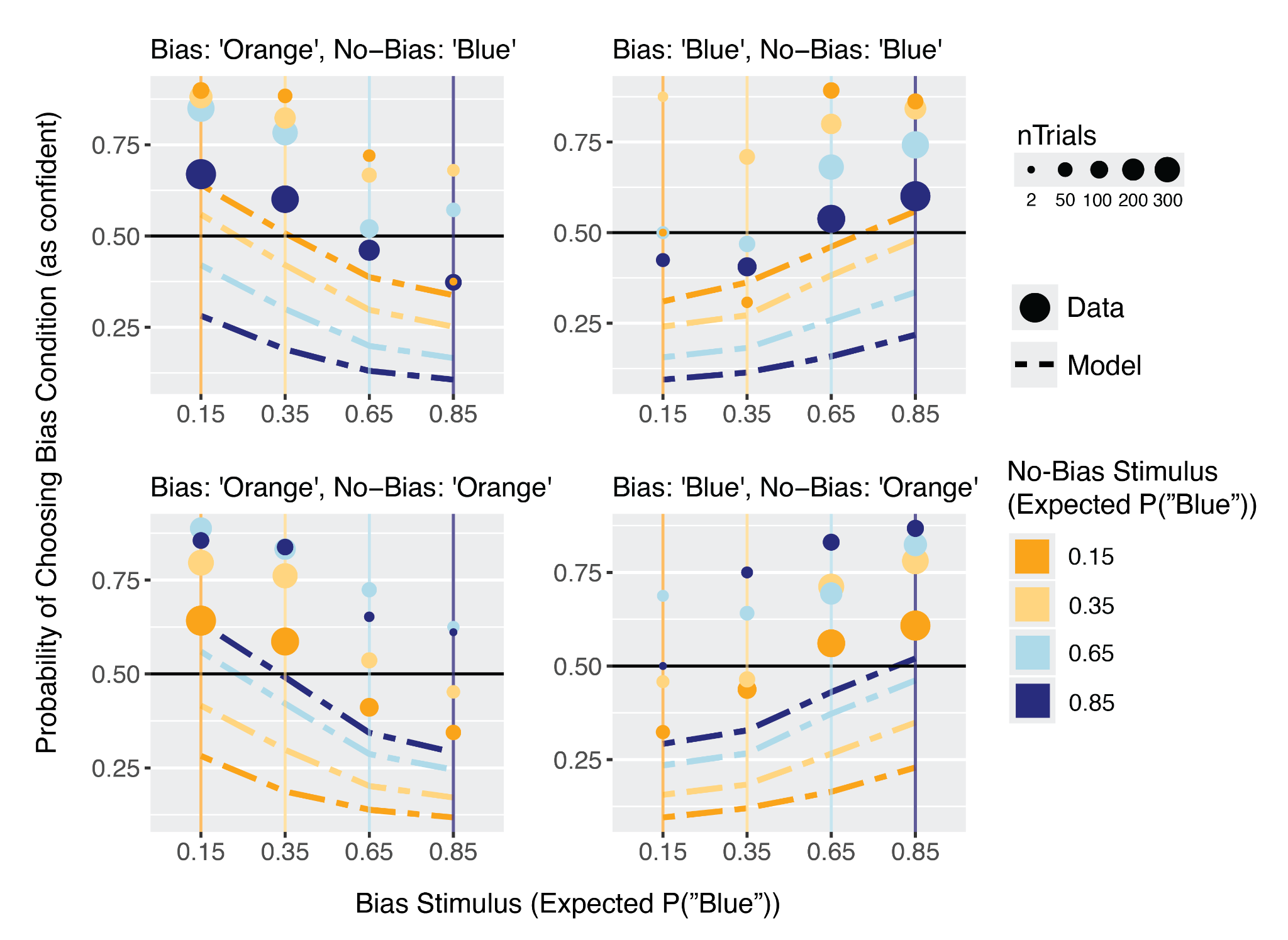


**Fig G. Confidence predictions from Condition-specific Likelihood (CL) model against data.** Confidence forced-choice predictions simulated from the CL model (dashed lines), which allowed the likelihood precision to differ between conditions, against the confidence data (points). This model still cannot account for the observed bias at the confidence level.

1. **CFC-model parameters**

**Table A.** Fitted confidence parameters from cfc-model (1). Confidence noise reflects inefficiencies in using sensory information to compute confidence (better confidence sensitivity for smaller confidence noise). Confidence boost reflects the fraction of new sensory information used to compute confidence above and beyond the information used for the perceptual decision (it varies between 0, for which confidence and perception have access to the same information, and 1, for which confidence uses completely new information, different from the one used by the perceptual decision). Confidence efficiency is an overall measure of confidence sensitivity that is normalized by the effects of sensory noise, similarly to the M-ratio measure (2). Interval bias reflects an overall bias to choose the first interval as the more confident one (negative values indicate that the second interval was preferred).

| **Parameter** | **Condition** | **Fitted Value** |
| --- | --- | --- |
| Confidence Noise, 𝜎_c_ | Bias | 1.07 |
|  | No-Bias | 0.54 |
| Confidence Boost, 𝛼 | Bias | 0.83 |
|  | No-Bias | 0.09 |
| Confidence Efficiency, 𝜂 | Bias | 0.78 |
|  | No-Bias | 0.59 |
| Interval Bias, 𝛾 | All | -0.26 |

1. **BIC results**


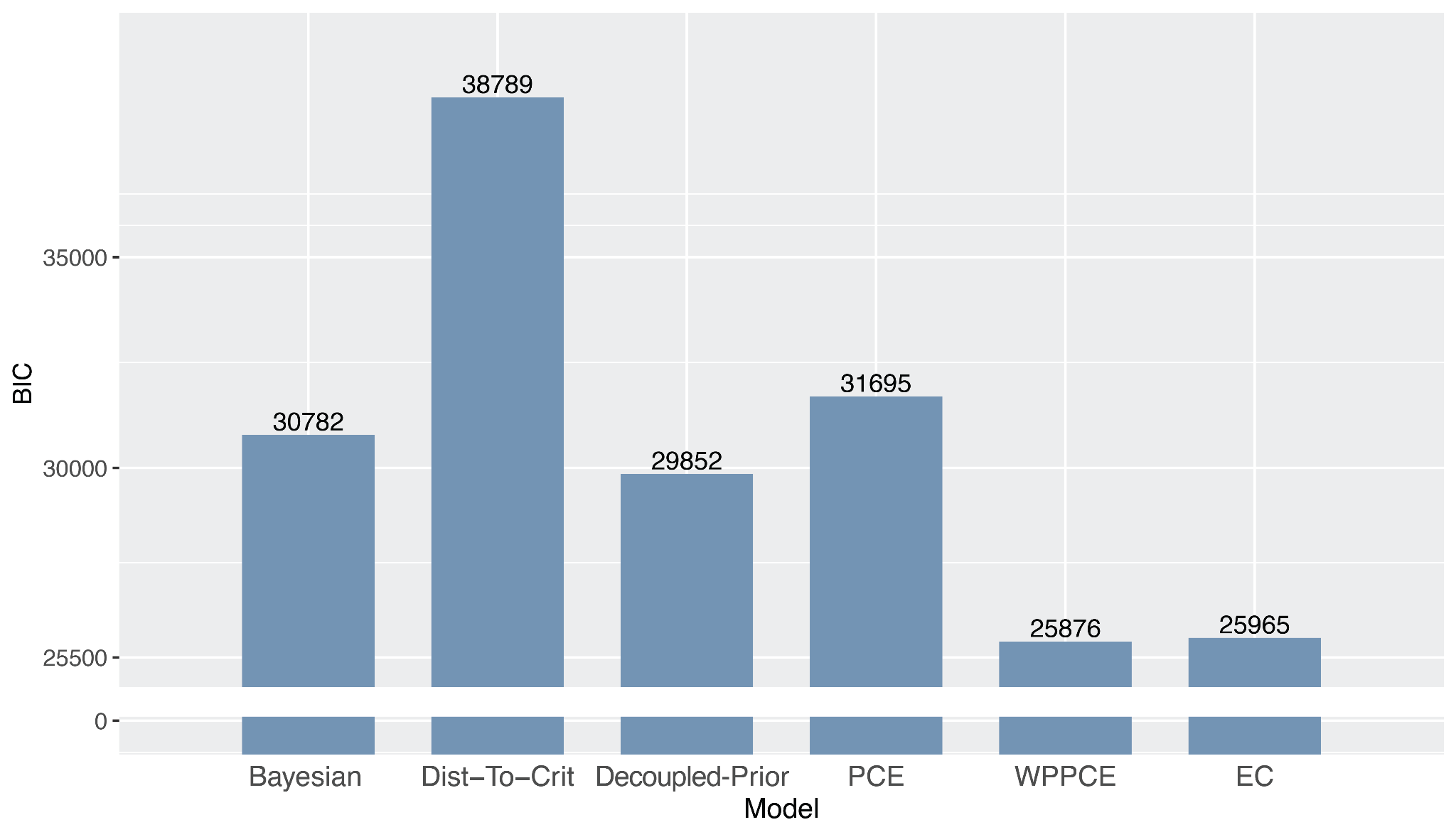


**Fig H. BIC group results.** BIC comparison of models fit to the pooled group data. As with the AIC results, WPPCE was found to be the winning model. We found a BIC_Bayesian_ = 30781.89, BIC_Distance-to-Criterion_ = 38788.65, BIC_DP_ = 29852.23, BIC_PCE_ = 31695.02, BIC_WPPCE_ = 25876.17, and BIC_EC_ = 25964.88.

1. **Model recovery analysis**

We ran a model recovery analysis in order to ensure that the models were adequately distinguishable in which we simulated data from each model and then checked that the correct model could be recovered. For each of 10 repetitions, we simulated 672 trials of data (equivalent to a single participant) from each model, using representative stimulus values by taking the mean stimulus intensity across participants for each expected performance setting. We also used representative sensory noise values by taking the median fit noise (σ_L_ and σ_P_) parameters across participants, and for the models with additional free parameters for confidence, we used the best fitting values from the group fits: *w*=1.54 for the Decoupled Prior (DP) Model and ɑ=0.34 for the WPPCE Model. For the EC Model, we used the median fit σ_P_, σ_N_, and σ_T_ parameters across participants. Models were compared using AIC, with the winning model captured as that with the lowest AIC value. Results of this analysis are shown in Fig I, revealing for each true generative model the proportion of repetitions for which each competing model was recovered as the winning one. This shows that the models were distinguishable from one another, successfully recovering the correct model in all but one repetition.


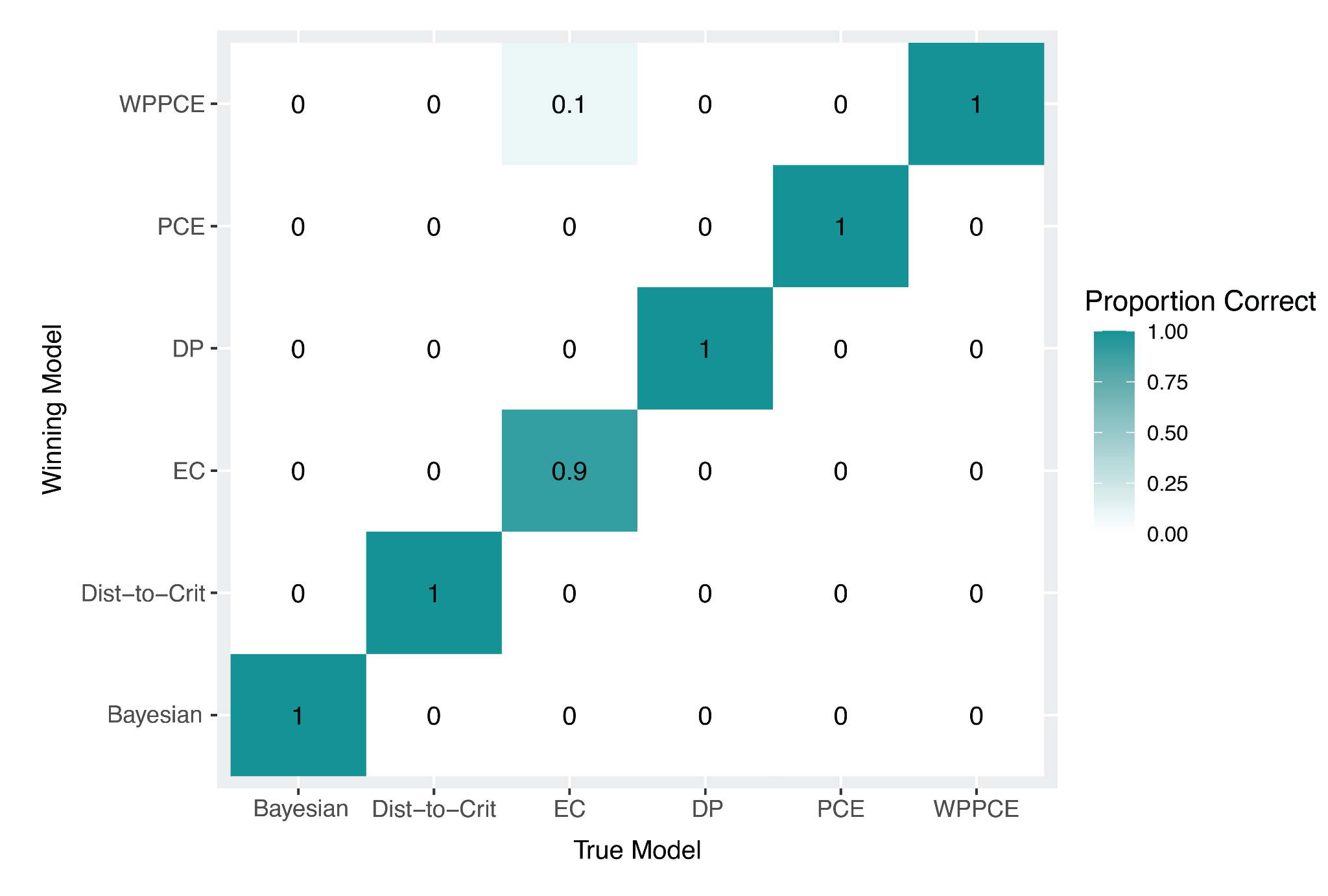


**Fig I. Model recovery analysis.** We tested model recovery by simulating data from each model, and then comparing each competing model’s fit to that data using AIC. Columns reflect the true generative model and rows reflect the recovered model. Cell color captures the proportion out of the 10 repetitions for which each competing model (row) was recovered as the winning one, given the true generative model (column). Cells diverging from the diagonal therefore indicate cases for which the incorrect model was recovered as the best fitting one.

**References**

1. Mamassian P, de Gardelle V. Modeling perceptual confidence and the confidence forced-choice paradigm. Psychol Rev. 2022;129(5):976–98.
2. Fleming SM, Lau HC. How to measure metacognition. Front Hum Neurosci [Internet]. 2014 [cited 2021 Feb 10];8. Available from: https://www.frontiersin.org/articles/10.3389/fnhum.2014.00443/full
