# Supplementary material for: Long-term perceptual priors drive confidence bias that favors prior-congruent evidence
Source: PLoS Comput Biol. 2025 Dec 22;21(12):e1013826. doi: 10.1371/journal.pcbi.1013826 (PMC12753060; doi:10.1371/journal.pcbi.1013826)
Supplement: Attachment — Submitted filename: Response to Reviewers.pdf [file pcbi.1013826.s002.pdf]

Dear Dr. Einhäuser,

Please find enclosed a revised version of our manuscript "Long-term perceptual priors drive confidence bias that favors prior-congruent evidence", by Marika Constant, Elisa Filevich, and Pascal Mamassian. We thank you for the opportunity to submit this revised version.

In response to the previous version, the reviewers made comments that we grouped into three main concerns, namely (1) that an alternative model might better explain the data, (2) that the Bayesian Optimal Observer model might be incomplete, and (3) about the WPPCE model being post hoc or limited. We now address these issues in key changes summarized here:

1. To address the possibility of an alternative model with an encoding asymmetry explaining the results, we included two new models capturing such an asymmetry: a 'Condition-specific likelihood' model that includes an asymmetry specifically across our conditions, and an 'Efficient coding' model that is more mechanistically complete and generalizable. Critically, neither model could capture the key confidence bias of interest, which is better explained as a confidence bias favoring prior-congruent evidence. Still, results from the new models align with research suggesting a neural efficient coding scheme, and provide tentative indications that efficient coding may influence confidence, even if it does not account for the confidence bias. To our knowledge this is the first work modeling confidence computations under an efficient coding scheme, so, – while not our main focus – it will provide an interesting approach for the field to build on.
2. While it is out of the scope of this work to consider all possible short-term priors that may influence a Bayesian observer, we now motivate in more depth our assumption of uniform task-related priors, and explain the experimental design choices we made to reduce the possibility of participants forming non-uniform task priors. We also soften the name of the model to remove reference to an Optimal Observer, and add several explanations of our particular approach: using a Bayesian model as a normative benchmark to assess the role of the long-term prior across processing levels.
3. We have clarified our modeling approach, highlighting the aim of capturing the observed bias effect rather than testing a set of *a priori* defined confidence models. We also better frame the logical steps towards the WPPCE, and add an exploratory correlation analysis to understand the use of the PCE cue in more depth. To address questions about the model performance, we have changed the format of our key modeling figures to better display the quality of the model fits and reveal the sparsely sampled regions in which performance previously looked poor. We also discuss remaining limitations and possible extensions to the model in a new section of Limitations, for future research to build on.

Outside of these main points, we have addressed other comments by more thoroughly and explicitly describing choices regarding the experimental design, analysis, and model implementations, thereby improving the clarity and reproducibility of the work.

We copy below the reviewers' comments and our responses (in blue). We would like to thank the reviewers for their comments, which we feel have helped us improve the manuscript. We would also like to thank you again for considering our manuscript for publication.

Sincerely,
Marika Constant, Elisa Filevich, and Pascal Mamassian

Reviewer's Responses to Questions

Reviewer #1: Review of "Long-term perceptual priors drive confidence bias that favors prior-congruent evidence"
Reviewer: Michael Landy

This is a nice paper that asks whether built-in long-term priors, which bias first-order estimates of stimulus properties, also have an effect on confidence judgments about first-order judgments. They cleverly do this with a prior that acts somewhat implicitly by using the slow-speed prior, which in addition to biasing speed estimate (e.g., for low-contrast stimuli), also biases motion-direction estimates from linear stimuli. Using a stimulus consisting of a set of short line segments in an aperture, they independently vary the orientation of the line segments and the motion direction of the line texture. When the line orientations are parallel to the motion direction, the tendency to see motion orthogonal to these contours is irrelevant, leading to no bias in motion-direction judgments. On the other hand, when the line orientations are close to orthogonal to the motion direction, perceived direction is biased toward the nearest orthogonal orientation of the line texture. The question is how will this bias affect confidence in judgments of motion direction. They do this using the lab's confidence forced-choice task and all possible (unequal) pairs drawn from 8 stimuli (4 are biased and 4 unbiased). They also propose and compare a variety of models of the behavior and find that judgments are best accounted for by a model that combines a Bayesian confidence with a second component that favors prior-contingent evidence.

All in all, I think this is an interesting and a strong paper. However, given my foggy memory, I just read it straight through and found that the exposition was needlessly confusing and could be made much easier on the reader in spots. The main thing I tripped on a bunch was what the conditions were, what the pairs were, and which pairs went into which analysis. Eventually, all of this is described somewhere, but if you read it from the beginning, it's easy to get confused. Why? Well for one thing, in several places it is stated that there are four bias-condition stimuli and four no-bias stimuli. But, there are also 4 levels of matched choice probabilities (.15, .35, .65, .85) and two kinds of biases (orange vs. blue). Thus, one might easily conclude that there must be 12 kinds of stimuli, not 8, and that maybe they are pooling some of them somehow. I only got un-confused when I save Figure 2A in which we find out that only two choice-probabilities are used for each of the two colors. Why? It's never said. I still don't know why you didn't include the other obvious two points on the blue and orange curves. Maybe, as in Locke, Gaffin-Cahn et al. (2020), those extra two conditions would lead to such biased choices that they wouldn't add any leverage to the modeling and would just increase the number of pairs (from 28 to 66) too much. It would be helpful to clarify this early and to motivate the choice.

> Thank you for these encouraging and useful overarching points. We agree that this could be clarified earlier in the manuscript, and we have now added lines 134-143 to do so. We now also motivate the choices in more depth in Methods (821-827).
>
> We wanted to have an equal number of trials in the Bias condition and No-Bias condition. Given that we have 4 No-Bias condition trial types, we needed to choose 4 Bias condition trial types out of the possible 8. We chose the ones for which the stimulus information (in terms of θ) would fall within the same range of extremity as the No-Bias trials, in order to (1) avoid having participants notice differences in the stimuli between the two conditions, and (2) avoid large conflicts between stimulus information and the prior, which may have additionally made participants more aware of their bias. So, we focused on using the conditions to contrast cases in which (a) the decision was driven just by the stimulus, versus (b) there was a bias favoring the decision and therefore the decision was

less driven by the stimulus itself. The earlier clarification of this (134-143) as well as further motivation of it (821-827) are copied here:

"In each condition, we used four θ values, targeting 0.15, 0.35, 0.65, and 0.85 probabilities of choosing blue (P('Blue')). The Bias condition could be set to induce a bias favoring either orange or blue, by either placing the preferred motion direction in the orange or blue region. However, we wanted to focus on the case in which the Bias condition required ***less*** *stimulus information than the No-Bias condition, due to the contribution of the prior. So, in the experiment, we did not include cases in which the bias worked against the stimulus. Therefore, when inducing a blue bias (with the preferred motion direction in the blue region), we only included θ values targeting 0.65 and 0.85 P('Blue'), and when inducing an orange bias (with the preferred motion direction in the orange region), we only included θ values targeting 0.15 and 0.35 P('Blue')."* (134-143)

"We did not include all four choice probabilities with both bias directions (blue and orange) because we did not want to examine cases in which the bias worked against the stimulus and hence the Bias condition actually required ***more*** *stimulus information than the No-Bias condition. Including those trials would have substantially increased the length of the experiment, and would have caused an imbalance in the number of trials across conditions, without adding much to our analyses. Such extreme Bias condition cases (in terms of θ) may also have risked the participants noticing the two conditions."* (821-827)

It's also the case that most of the analyses drop a bunch of the pairs, only including pairs that have one bias and one no-bias stimulus in them. Why didn't simply only run those pairs? And tell us early on that you are dropping them, please.

We also included pairs with the same condition in the experiment as these helped with the fitting of the cfc-model. Later analyses required only the pairs that have one bias and one no-bias stimulus, and we now state more clearly when we drop these (273-274; 386-387) and state this choice more explicitly in Methods (899-902):

"Trials with the same condition in both intervals were also primarily included to constrain the fitting of the cfc-model, and were removed from several of the other analyses, such as the non-parametric confidence analysis and the modeling building on the Bayesian model."

Other comments:

Line 41: "are combined with weights proportional to their precision": this is tacitly assuming Gaussian noise if you are talking about a Bayesian model. You know this, but I like things explicit.

We now state this explicitly (41).

Lots of places: You randomize the reference orientation and avoid the cardinals. This effectively averages out any cardinal-direction priors. I was surprised you never discussed this explicitly.

True, we intentionally did this to remove the possible influence of cardinal orientation priors on the results. We now state this explicitly (846-848).

Figure 1A: This is the only place \lambda is defined or used. For example, line 838 doesn't use \lambda.

We have now removed reference to lambda in the Figure, as it is not very helpful to refer to it elsewhere.

144: "The strength of the likelihood" is a weird phrase. I'm not sure what aspect of the likelihood is meant (it's width???). Also, it's not completely controlled by \theta, because that's the stimulus orientation (relative to the reference), which differs from the noisy measurement, and it's the lateral upon which the likelihood is based.

We agree that this is unclear and have now clarified this phrasing (152). We are really referring to the discrimination difficulty, in terms of the distance between the true motion direction and the reference, which will impact the *mean* of the likelihood distribution.

157: Please indicate the prior direction \lambda = 35 for the blue and orange bias, either in the figure caption, in the main text (line 175), or both.

We now indicate this both in the figure caption and main text (183-184).

198: "intensity"???

This was an error and has now been removed. We now refer to stimulus difficulty (208).

282: In some ways this is the bottom line of the paper, but it's a pretty modest (12%) effect.

We contend that this is not a modest effect, especially considering that it captures only the *dissociation* between the influence of the prior at the Type 1 and confidence level, rather than the overall effect of the prior on confidence, which is much larger. This is now clarified in the text (293-295). Further, the effect is very consistent across participants in terms of the direction of the bias (see Appendix S3 for subjectwise fits), and is enough to qualitatively change confidence patterns across different stimulus combinations, so it will have a relevant impact on confidence predictions. Also, the effect is even stronger if considered in light of the Bayesian model, which predicts an effect in the opposite direction.

217: Why do you allow sensitivity to vary across conditions here, while using a constant sensitivity in the manipulation check task? Additionally, individual fitting results should be included in the appendix.

We note that we did not use a constant sensitivity for the manipulation check from the ASA procedures either, but allowed sensitivity to differ between conditions. We now state this clearly (178-179).

We allow sensitivity to vary across conditions here because this modeling step was focussed on naively quantifying behaviour, and it is possible that the prior impacted overall sensitivity. We now state this (226-227) and include individual fitting results in the Appendix (S2). In contrast, the Bayesian model in later analyses captures the precision of the prior and likelihood separately, so for that we assumed, on conceptual grounds, that the likelihood would have equal precision across conditions (though note that we now include a Bayesian model that allows different likelihood precisions as well, in response to several other reviewer comments).

227: It actually makes sense that the choices do not match the estimates from the manipulation check task. In the manipulation check, the prior effect might be stronger because all trials in a staircase block are either

biased or unbiased, whereas the main task trials are intermixed. At least the Methods' description of the manipulation check seems to imply the conditions aren't interleaved. Otherwise, clarify the Methods.

> They were interleaved in the ASA procedure as well, which we have now clarified in Methods (811-812). Also, the prior effect actually seems to be stronger in the experiment, which we interpret as participants getting tired, hence the likelihood getting noisier and the influence of the prior getting stronger.

Figure 2B: The x-axis label here is incorrect (and the tick marks make that obvious). As in the running text, this is the difference of proportions first turned into z-scores.

> Thank you for catching this, we have now fixed it (although note that they are also now transformed using a logit function rather than z-scores, in response to Reviewer 2).

Fig. 3A: The 2nd row is labeled "Stimulus", but the gray curve is a measurement distribution for a fixed stimulus.

> We now label this Sensory Measurement.

284-301: A more detailed description of the CFC model is needed, at least in the appendix, as the parameter estimates are provided in the appendix without sufficient explanation of the model.

> We now include more explanation of the cfc-model in the Methods (924-941) as well as in the Appendix S6, before providing the parameter estimates.

307: Maybe you should say 35 deg/ -145 deg?

> We have fixed this.

408: "a single sample from the posterior": This doesn't seem feasible. The posteriors here are pretty complex, so how would an observer sample from it? And, the sample can be on the opposite side of the criterion compared to the participant's first-order response, which is weird. Why not use MAP or MEAN of the posterior instead? This is much simpler than sampling, and you have no evidence of sampling. Just because you don't ask for an estimate doesn't mean they don't infer one.

> It is not clear to us how participants could easily compute the MAP or MEAN without knowledge of the full posterior distribution. We agree that it is possible that they infer a mean estimate even though we don’t ask for one, but we wanted to keep this model as close as possible to other confidence models that base confidence on the distance between a sample of internal evidence and the criterion. The model also assumes that the sample of posterior evidence cannot be on the opposite side of the criterion compared to a participant’s first-order response, as we agree this would be unintuitive. We consider confidence choice rates from simulations that lead to the appropriate pair of first-order decisions. Thank you for pointing out that we had not stated that. It has now been included (433-435; 1013-1015).

Figures 4 and later: These 4x4 colored figures: Are the data from pooling over participants? Didn't the participants get participant-specific stimuli chosen based on the control experiment? And wasn't that experiment unsuccessful in matching choice probabilities? As such, isn't it problematic to pool them in these categories, since the actual choice probabilities came out differently for different participants?

Note that we have now changed the format of these 4x4 colored figures, but we think the comment still applies to the new format, so we respond to it in light of the new figures.

Yes, it is true that the participants got specific stimuli that were not perfectly successful at targeting matched choice probabilities, despite being close. It is also true that these figures show the pooled confidence data grouped by the *targeted* choice probabilities, not the actual ones, and this might include slightly different actual choice probabilities across participants. However, despite the fact that the figures are grouped by targeted choice probabilities, the *actual* choice probabilities for each individual participant were used to fit and simulate from the models. Therefore, the model results for each category in these figures reflect the same set of actual choice probabilities as the data, and are hence directly comparable to the data points. The new figure format means that the model and data are now also shown in the same figures, for ease of comparison. We have clarified this in the description of the new figure format (403-407).

502: the "true motion direction" isn't known to the participant, so this vector projection should be based on a noisy measurement. Maybe the average will come out this way as plotted though in the expected value. Not immediately clear to me.

We completely agree, and the vector projection in the model is indeed based on a noisy measurement of the stimulus. The figure is just to convey the intuition, but this has now been clarified in the text (517-518), and figure caption (529-530).

WPPCE model: This left a bad taste for me. You have a bunch of models. None work. But one is oriented vertically and one diagonally and the data look in between, so you took a weighted combination of failing models to get one that fit the data. Hmmmm.

We understand that the current WPPCE model can come across as a post-hoc solution. The way we view it is that we considered the different possible sources of evidence which could plausibly explain the effect of interest, namely, the confidence bias favoring the condition with more influence of the prior. This effect was already clear from the model-free analysis and our modelling was targeted at explaining it, rather than testing a set of confidence models defined a priori. The Bayesian Optimal Observer model was our starting point and normative benchmark, as it is in line with the widely used Bayesian confidence model and intuitively supposes that participants base their confidence on the same evidence used in the perceptual decision. When this was insufficient to capture the effect of interest, we considered specific model deviations from the Bayesian benchmark that might capture the observed confidence bias, such as an overweighting of prior evidence (WP model) or the use of other prior-related cues that might be available to participants. This is the context under which we considered the prior-congruent evidence (PCE) in the stimulus: we supposed that it might be a plausible additional source of evidence that participants use, which could potentially explain the observed bias. The PCE model demonstrates the extreme case of using *only* this cue for confidence. However, we agree that it may be somewhat misleading to frame this as a stand-alone confidence model because, while possible, we did not realistically expect that this source of evidence would alone account for confidence judgments. We are now more explicit about this (514-516; 522-524). Instead, our intuition was that it might serve as an additional cue to confidence, which is captured in the WPPCE model. The approach of the WPPCE also fits with similar confidence models in the literature that consider different sources of evidence or cues that might influence confidence beyond what is used in the decision, such as the weighted evidence and visibility model which similarly takes a weighted combination of the posterior evidence and cues regarding the stimulus visibility.

We have rewritten the framing of these modeling steps (between 506-561) to be more explicit about the approach of formalizing the possible sources of evidence and the steps towards their combination in the WPPCE. We clarify that the inclusion of the PCE model is primarily for completeness and for the demonstration of how this cue impacts confidence (522-524). We also highlight our attempt to capture the confidence bias that we see from the model-free analysis in our model considerations, rather than consider a priori a set of possible confidence models that could occur in this task (313-316).

Here's another idea, perhaps equally distasteful, but that might make similar predictions: Have the prior depend on the task, as follows. The prior could be a Gaussian or a von Mises. Let the concentration parameter of the prior depend on \lambda, for example:

\kappa(\lambda) = \kappa_0 | \sin(2\lambda) |

This predicts increased bias in the bias condition because the prior not only shifts the posterior mean but also makes the posterior narrower, so there's more probability density on one side of the criterion. In the no-bias condition, the posterior would be equivalent to the likelihood and wider than in the bias condition.

This is an interesting idea, but to have a prior whose precision changes based on the relationship between the line orientation and an arbitrary, task-specific reference goes against the standard notion of priors – particularly such a low level perceptual prior – and does not seem easily justified. However, we believe this is similar conceptually and makes similar predictions to a model in which the precision of the *likelihood* depends on the stimulus, with stimuli that are more probable (moving in the preferred motion directions) being encoded more precisely. This is in line with the idea of efficient coding, which there is support for in the literature. We now implement and include an efficient coding model (574-595; Figure 7), which is described in more detail below, in response to Reviewer 2.

701: stimulus "values?". I presume you mean texture orientations, but please say so.

We meant that we selected the θ values (stimulus difficulties) to target the desired choice rates, which we have now stated explicitly (801-803). The texture orientations were sampled uniformly for all participants.

706 or so: State explicitly that the staircase controlled motion direction.

We now explicitly state this (809).

712: Why no lapse rate?

From piloting, lapse rates were low – we think in part due to the cursor-based response format with large response regions, which at least reduced motor lapses – and adding a lapse parameter rarely improved the fits, but sometimes caused problems in the model fitting. As we did this fitting during the actual experimental session in order to select stimulus settings for the full experiment, we needed it to run quite automatically and robustly, and we found that the costs of including lapse outweighed the benefits. We now mention this in Methods (816-817).

727: Why 3.5% here when it was 3% before?

The 3% was only part of a short stimulus demo aimed only at showing participants what the stimuli could look like at both extremes of a range of difficulties, but indeed was even slightly more extreme than the contrast used for the full experiment. 3% was, however, only ever used in the short demo, during which responses were not recorded. All data from all stages of the experiment used a contrast of 3.5%.

751: 2.86 deg of visual angle IN LENGTH

We added this (871).

765: This isn't the kind of stimulus for which I'd typically use Michelson contrast since most pixels were in the background. I'd report Weber contrast (i.e., 6%).

We adjusted this to report Weber contrast.

773: "cursor was invisible": Did it start out visible or did they know somehow that it started in the center? How else would they know where to move it?

Yes, they were told that it always started in the center and had an opportunity to get used to this in the stimulus demo. We have clarified this (884-885).

782: I can't parse this sentence. What specific stimulus settings had no bias effect and how did you know that???

We agree that this is unclear and have rewritten it to clarify (892-899). For some participants, the expected perceptual effect of the prior – being biased towards the preferred motion direction – was not there. This was likely due to interindividual variability in terms of both the strength of the slow-motion prior and internal noise (and hence the likelihood), leading to some participants requiring lower contrast in order for the bias to emerge. We chose to keep contrast constant across participants at the risk that for some, the bias would be too strong, and for some, the bias may not appear at all. Because of this, and because our hypotheses focussed on comparing the confidence level to the Type 1 level, rather than exploring this basic perceptual effect, we preregistered that we would exclude data from cases that did not demonstrate this perceptual bias to any extent. For data to meet this criterion, we required that the θ value chosen to target a given choice probability (from the ASA procedure) in the No-Bias condition was more extreme than the θ value targeting the same choice probability in the Bias condition. This indicates that the prior contributed to the Bias condition choices to some extent, demonstrating the basic bias effect.

797: I think that, at least in Methods, you haven't yet said that you are omitting trials that aren't one each of bias/no-bias in the pair, but the wording requires that to be true.

We have now clarified this (899-902; 914-915).

816: into one psychometric FUNCTION

We have added this (943).

838: Should you state that these were circular Gaussians (that wrapped around) or their sigmas were low enough so that wraparound wasn't an issue?

This was not an issue due to the low sigmas and the fact that the choice region over which we integrated was always only between -90° and 90°, but we have mentioned this now (966-968).

892: It's weird to call this a "weight" when it's an SD and this isn't the Gaussian case so it doesn't end up acting (indirectly through precision) like a weight. I'll note, FWIW, that my lab has a paper (in press, Proc Royal Soc B) that claims that, in fact, people use an SD for inference that is smaller than the one we experimenters estimate from discrimination. But you folks already know that ;^)

We agree that it might be somewhat misleading to call this a weight. We did this to emphasize the change in the precision of the likelihood relative to the prior between the Type-1 and Type-2 level. But, we changed the name of this model to the Decoupled Prior Confidence Model, and adjusted the descriptions of it (pg. 16-17 and 34-35).

The link to your Proc Royal Soc B paper is very interesting (now included in the references). In a certain sense it may go in the opposite direction of our findings, in which participants essentially seem to use a larger SD for the likelihood (relative to the prior) in their confidence inferences compared to what we estimate from their discrimination. However, in our case this is always *relative to the prior*, so it is difficult to compare the results directly. We now mention the possible relationship between these findings (476-478). But, this is not the winning model so in our case we think we are capturing a separate effect.

915: There's no reason to include "radius" in this formula

We agree and have removed it.

Table S5: I'm glad you include fitted parameters (based on pooled data???). But, there are some big effects here (difference in confidence noise and in confidence boost) that you never discuss. Why not?

The data were indeed pooled in order to give us enough trials for fitting the cfc model, but we used normalized stimulus values per participant in order to pool them. We fit the cfc model in order to give us an additional quantification of the confidence bias before implementing the Bayesian model. However, because we expect that the prior favoring orthogonal motion directions will be non-Gaussian, we also expect a non-Gaussian posterior. So, we wanted to avoid interpreting the results of this model too strongly in isolation. We also did not thoroughly explore how reliably confidence noise and boost could be estimated given our data, as this was not our research question and was out of the scope of this work, but we report these parameters here in case somebody would like to perform a meta-analysis in the future.

Reviewer #2: This paper investigates the role of long-term perceptual priors on confidence in perceptual decision making using a Bayesian framework. The authors manipulated their stimuli so that a natural prior favoring slow moving stimuli as explanations for percepts either informed perceptual decisions or was orthogonal to the decision at hand. They find that confidence is higher in decisions where the natural prior induced a perceptual bias. Based on this finding, the authors claim that confidence relies more on evidence that confirms existing beliefs (i.e., is informed by the natural prior). In my opinion this is an interesting study but its conclusions are not fully supported by the approach. The data and model need to be scrutinized further before I would find this acceptable for publication. I outline this below.

We thank the reviewer for their careful reading of our manuscript and for the detailed propositions of alternative interpretations.

Major concern:
What the authors call the "Bayesian Optimal Observer Model" is too simplistic to draw the kinds of conclusions that the authors want to draw. The confidence in a judgement is directly related to the posterior probability of that judgement being correct. The posteriors of the bias and no-bias conditions are assumed to differ only because of the prior, while the likelihoods are assumed to be equal, but that is not properly motivated in my opinion. The long-term prior for lower speeds likely also biases the encoding and therefore affects the likelihood (e.g., https://doi.org/10.1038/nn.4105). The model formulation as presented assumes that the internal noise is independent of the angle between line orientation and line motion direction, but this is clearly not supported by previous data (https://doi.org/10.1167/11.3.25) and seems to be unsupported by the presented data. This suggests that the sensory measurements in the Bias condition were less noisy than the sensory measurements in the No-Bias condition, which naturally explains the difference in confidence.

This might be mitigated somewhat by the attempt to match the proportion of 'blue' and 'orange' choices in Bias and No-Bias conditions. Since different values of motion directions were used for the Bias and No-Bias conditions (line 185), it is not clear how big this difference in encoding precision between Bias and No-Bias conditions should be. The data however seem to show that participants were more sensitive in the bias condition (Fig 2), and they seem to have been more accurate at picking blue and orange when the stimuli were blue or orange respectively (lines 196-220). This is easily explained by varying the internal noise based on the angle between the line's orientation and its motion direction.

While we agree that there is a possibility of an encoding asymmetry such that the likelihood precision is itself biased by the prior, our design aimed to match the *posterior* information across conditions by matching the first-order choice rates, as the reviewer also acknowledges, which could in principle account for differences in likelihood precision. The idea was that any remaining confidence bias would then reflect different information entering into the confidence decisions compared to the perceptual decisions. However, to explore the possibility of differences in likelihood precision due to the prior, we now include two new models.

First, we include a version of the Bayesian observer model which allows for a different likelihood precision in each condition. This primarily serves to investigate whether a naively implemented encoding asymmetry across conditions can already account for the observed confidence bias. We fit this model to the perceptual decisions of each participant, which revealed a median likelihood noise in the Bias condition (σ_{L-Bias}) of 19.01, and in the No-Bias condition ($\sigma_{L-NoBias}$) of 20.62. The lower variance in the Bias condition, though slight, is consistent with the possibility that the sensory encoding was more precise when the stimulus was in line with the prior. However, when we simulated the confidence patterns of an observer that uses the same information in confidence choices as in perceptual decisions, even with the more precisely encoded Bias condition information, this model could not capture the observed confidence bias favoring the Bias condition. The results from this 'Condition-specific Likelihood' model are now included in the Appendix (S5) and referred to in lines 591-593 of the main text.

Next, although the Condition-specific Likelihood model did not explain our pattern of results, it did reveal some evidence in support of such an encoding asymmetry, and we agree with the reviewer that this is consistent with other findings in the literature and warrants further investigation. So, we

implemented a full efficient coding model, following the approach of Wei and Stocker (2012,2015). The details of the model definition are described in Methods (1065-1083) and copied here:

“The efficient coding idea suggests that the brain should allocate more resources to represent stimuli that are more likely to occur. The implementation of efficient coding in the model here follows that of Wei and Stocker (37,63). They formalize the link between allocated resources – as measured by the Fisher information of the sensory evidence, J(θ), – and the stimulus probability – the prior, p(θ), – such that $p(\theta) \propto \sqrt{J(\theta)}$ *. A function F(θ), which is defined as the cumulative of the prior, maps the physical stimulus (θ) to a sensory space with uniform Fisher information, yielding* $\tilde{\theta}$*. We then fully define the likelihood distribution in this sensory space by assuming Gaussian neural noise of standard deviation* σ_N*, and a mean that is jittered from* $\tilde{\theta}$ *due to transduction noise,* σ_T*. The likelihood in physical space is obtained using the inverse mapping F-1(*$\tilde{\theta}$*). The resulting likelihood will not be homogeneous in physical stimulus space and will often be asymmetrical (Fig. 7A). All other computations are identical to the Bayesian model (Eq. 1-4), but using this differently defined likelihood. This means that the likelihood, which is already constrained by the prior, then gets combined with the prior in the Bayesian integration to form the posterior, effectively giving an additional impact of the prior on processing.”*

We fit this model to the perceptual decisions of each individual participant and simulated confidence patterns from it, in order to investigate whether such an efficient encoding scheme could explain the observed confidence bias. This revealed that it could not: it still underpredicted the confidence choice rates favoring the Bias condition (new Figure 7). Further, the WPPCE was found to be a better model in the model comparison (AIC difference of 89). But, interestingly, the efficient coding model was found to be a better model than the Bayesian model without efficient coding. Together, we take this as tentative evidence that there is efficient coding, consistent with previous findings in the literature, and, further, that this efficient coding influences confidence (which has not been previously investigated to our knowledge). But, this efficient coding does *not* explain the dissociation that we observe between perceptual decisions and confidence, with confidence still being disproportionately higher in the Bias condition. Instead, this is better explained by prior-congruent evidence biasing confidence.

Besides the details in Methods, copied above, we have added a description of this model and the results from it in Results (574-595; Figure 7). It is also now included in the model comparison and model recovery analysis.

Additionally, there are more sources of prior information available to the participant than just the orientation of the moving lines. The long-term prior that is manipulated by the authors (i.e., the line orientation) has to be combined with a shorter-term prior about the structure of the experiment. It is not immediately clear how this prior should be modeled, since participants received no feedback about their answers and so might have had uncalibrated assumptions about how correct their answers were. Whether the shape of this task prior is uniform or gaussian or something else is an open question. Without considering this task-related short-term prior, the “optimal Bayesian observer” is incomplete.

We agree with the reviewer that our task is too under-constrained to be in a position to know exactly what priors were used by our participants. We avoided providing feedback because we did not want participants to learn a particular mapping between stimulus, perceptual decision, and confidence judgment. While these learning experiments are very interesting on their own, our interest here was simply to measure the default behavior of participants. We also wanted to avoid participants

becoming aware of their perceptual bias, and providing feedback would have risked that. Although this limits our ability to model the learning of task-related structure, our experimental design aimed in several ways at reducing the possibility of any non-uniform task-priors, which we highlight below.

First, participants' goal here was not to infer which stimulus they had encountered out of a set of possible stimuli, but rather to perform a continuous perceptual estimation task which we crudely mapped onto a discrete decision by having them compare their percept to a reference. Hence, we took the simplest assumption that their task prior was uniform over the possible -90° to 90° (relative to the reference) range of motion directions. Additionally, the experiment was designed to discourage participants from noticing other structure: the line orientations were sampled uniformly trial to trial (though excluding cardinal orientations), and both the reference placement and motion direction were set on each trial on the basis of that orientation. This means that, though there were 8 fixed stimulus difficulties in terms of theta – the distance *between* motion direction and reference – the actual absolute motion directions encountered around the circle varied much more uniformly across the entire 360° range. We also counterbalanced whether the orange or blue option was the clockwise one in the response region, and this changed trial-by-trial. So, taken together, the absolute line orientations, reference placement, motion direction, and placement of orange and blue response regions varied unpredictably on each trial. Further, the reference, response regions, and line textures all appeared at the same time, and the motion occurred immediately. This prevents participants having time to form short-term priors about the motion direction *relative* to the reference. To avoid short-term priors forming on the basis of the decisions themselves, the Bias condition had a bias favoring blue half of the time and orange half of the time, and these were randomly interleaved. So, there should have been no noticeable bias in the overall choice rate favoring orange or blue responses. And, the confidence forced-choice task structure added even more diversity to the task, with two perceptual decisions for every confidence judgment, in pairs that were balanced in terms of the pairs of expected answers. All together, this made it very difficult for participants to form short-term priors about the task structure beyond just a uniform prior.

That being said, we agree that it is still possible that participants formed short-term priors about the task structure, and without considering them, it is misleading to call this an Optimal Bayesian Observer model. It is out of the scope of this work to model all possible short-term priors one could form, and it is indeed unclear how they would form these. As such, we soften the name of the model used to just the Bayesian model, and have removed mentions of modeling an optimal Bayesian observer. We also highlight our specific focus on the Bayesian use of the slow-motion prior when framing the model (313-316). The limitations identified here are also discussed in more detail in the Limitations section of the manuscript (735-742), and these lines are additionally copied in response to Reviewer 3.

There is a chance that a proper ideal Bayesian observer model that includes all available prior information and an encoding asymmetry, where the precision of the likelihood varies with the angle between line orientation and motion direction, explains the data by itself.

The authors need to show either that a Optimal Bayesian Observer model that correctly accounts for the sensory noise does not reproduce the higher confidence in the Bias condition, or they need to show that the sensory noise (i.e., the precision of the likelihoods) does not change when the angle between line orientation and motion direction changes. Note that I don't mean theta, the angle between reference and motion direction, but rather the angle between the line orientation and the motion direction, which is how the long-term prior is manipulated.

We have now included an efficient coding model (described above) that implements the idea suggested by the reviewer – the likelihood is itself constrained by the prior in such a way that its precision changes when the angle between line orientation and motion direction changes. This model still cannot capture the confidence bias observed favoring the Bias condition, and does not outperform the WPPCE model.

This seems conceptually similar to scaling the variance of the likelihood like in the Weighted Prior Confidence Model that the authors propose. However, in that model all Bias likelihoods get scaled by the same constant. If the sensory noise depended on the angle between line orientation and line motion direction, different conditions (e.g., P(“blue”)=0.15 and P(“blue”)=0.35) would be scaled differently. This difference should allow the authors to differentiate between the Optimal Bayesian Observer model and the Weighted Prior Confidence Model.

We thank the reviewer for this helpful suggestion. We implemented an efficient encoding model following the formalization of Wei and Stocker rather than using a scaling function as an approximation, but the result is in line with what the reviewer describes and is indeed consistently differentiable from the Weighted Prior model. This is confirmed in the model recovery analysis, in which the efficient coding model is now included (Appendix S8).

If it turns out that stimuli with more prior-congruent evidence lead to less variable measurements (as suggested by https://doi.org/10.1167/11.3.25), then the conclusions from this experiment should be very different from what the discussion presents at the moment. It would suggest that there is no confirmation bias in those confidence judgements at all.

Minor concerns:
- Some imprecise language should probably be cleaned up, for instance “strength of the likelihood” (line 134) probably means “precision of the likelihood”.

We have adjusted this language to be more precise, also in accordance with comments from Reviewer 1.

- Z-scoring of proportions that are bounded between 0 and 1 (lines 266-267) is not appropriate. The authors could use logit (log(p/(1-p))) or Fisher’s arctanh transformation instead or they might pick a different function to fit to the raw data, so that a transformation is unnecessary.

We have now changed this and use the logit transformation.

Reviewer #3: In this paper, the authors examined how long-term perceptual priors influence confidence judgments, using the slow-motion prior in motion perception. Participants were presented with pairs of tilted moving-line stimuli and made motion direction judgments (which the author categorized as blue vs. orange region) followed by a confidence forced-choice to decide which of the two intervals they were more confident in. The authors designed two conditions: a Bias condition, where the slow-motion prior systematically influenced perceived direction (thus favoring one response), and a No-Bias condition, where the prior had no directional influence. The key finding was that participants showed a confidence bias favoring the Bias condition, even after accounting for performance differences. Through computational

modeling, they discovered this effect was best explained by a model where confidence uses both Bayesian posterior evidence (used in perceptual decisions) and prior-congruent evidence as an additional cue. They concluded that long-term perceptual priors, like previously studied high-level expectations, have a greater influence on metacognitive confidence than on perceptual decisions themselves.
The manuscript is overall well written, the experiments are carefully designed and well executed, and the topic addresses an important question in the field of perceptual decision-making and metacognition. The study contributes meaningfully to our understanding of how long-term priors shape confidence independently from decision accuracy and will likely be of interest to researchers working on Bayesian models of perception and confidence. However, I have several concerns regarding the modeling assumptions and analytical approach. These issues are detailed below.

We thank the reviewer for the positive feedback, as well as the insightful considerations regarding the modeling and analyses, which we discuss below.

Major comments

1. The authors implemented a Bayesian optimal observer model assuming that participants had perfect knowledge of the generative structure of the task, which would include the difficulty level in each condition (the “targeted fraction of choice”). However, it seems that participants were never explicitly informed of these conditions, and it is unclear whether they could have reliably inferred the statistical structure. Thus, given that the subjects can only infer the actual statistical state of the environment but not have a full accurate knowledge of it, I wonder whether it is fair to frame this as a test of the Bayesian Confidence Hypothesis (BCH) in its strictest sense (Li & Ma, 2020; Xue et al., 2024). Maybe it is worth to more carefully consider what task structure information was available to participants and discuss whether the model’s assumptions are reasonable representations of participants’ internal knowledge. In other words, the observed deviations from optimal Bayesian behavior might simply reflect incomplete knowledge rather than fundamentally different computational processes.

Our focus was not to test the Bayesian Confidence Hypothesis, but rather to specifically assess the influence of an informative, long-term prior in confidence, making use of a Bayesian model to do so. We have adjusted the framing of the model to emphasize our focus on capturing the use of the slow-motion prior (313-316; 740-741), and elaborate on how our approach fits the literature testing the BCH (also in response to the reviewer’s minor comment below; 701-717). So, we agree that the task was not structured to test the BCH in this strict sense, and, in fact, there were critical differences between our experimental design and those aimed at capturing the learning of task-related priors. Here, participants' goal was not to infer the statistical structure of the task. They did not need to infer which of a set number of stimuli the evidence came from, but rather performed a continuous estimation task of the motion direction, which we then very crudely discretized by having them compare their percept to a reference. As such, in our model, we do not assume participants have any knowledge of the task structure (including the difficulty levels), but rather model it as a continuous estimation task of the motion direction (with a uniform prior), discretized by comparison to the reference. In other words, we assume a uniform task-related prior and, while we agree that our task is too underconstrained to know exactly what structure participants inferred, we made several design choices to reduce the possibility of them forming non-uniform task priors, highlighted below:

The line orientations were sampled uniformly trial to trial (though excluding cardinal orientations), and the reference placement and motion direction were set on each trial on the basis of that

orientation. This means that, though there were 8 fixed stimulus difficulties in terms of theta – the distance *between* motion direction and reference – the actual absolute motion directions encountered around the circle varied much more uniformly. So, the line orientation, reference placement, and motion direction all varied almost uniformly across the full 360° range trial by trial. We also counterbalanced whether the orange or blue option was the clockwise one. And, the confidence forced-choice task structure added even more diversity, with two perceptual decisions for every confidence judgment. Together, we believe this made it very difficult for participants to form short-term priors about the task structure beyond just a uniform prior.

However, because it is true that we are not testing the range of possible task-related priors that participants could form – as this would be out of the scope of this work –, we soften the name of our model to the 'Bayesian model'. We also discuss this directly in the Limitations section of manuscript (735-742), copied here:

"It is also possible that participants formed other short-term priors about the task structure. We tried to avoid this by having the line orientations and absolute motion directions sampled nearly uniformly across the full 360° range, making conditions and stimulus settings difficult to detect. But, future work could use a more conspicuous task structure and actually model participants' learning of it. This would make a more complete Bayesian Optimal Observer model, where here in our Bayesian model we focus specifically on a Bayesian use of the slow-motion prior in decisions and confidence."

2. Another way I can think of to model the prior is to model it within a signal detection framework, and in such priors are typically modeled as a shift in the decision criterion (Green & Swets, 1966; Zheng et al., 2024). Under this framework, when making decisions aligned with the prior, subjects would naturally have higher confidence due to the greater distance between the stimulus evidence and the shifted criterion. Even when making a decision not aligned with the prior, it would not result in a higher confidence, like the prediction of the weighted prior model. This happened because the criterion shift means that the distance between the prior-congruent category and the decision criterion is larger. I wonder what the authors' thoughts are on this.

This is indeed interesting to consider, and many of the predictions of the Bayesian model can be mapped into this signal detection framework. However, in our task there is no explicit sensory criterion, since the reference moved freely from trial to trial. We also counterbalanced and randomly interleaved trials with the bias favoring orange versus blue (and clockwise versus counterclockwise), so there was no overall bias formed across trials even in the Bias condition. Further, there was no visible indication to participants about whether a given trial was in the Bias or No-Bias condition, or what direction the bias would be if there was one. So, it is difficult to see how a criterion shift could be an appropriate explanation in our task. Additionally, although there is no overall decision bias in our No-Bias condition, we still expect a *perceptual bias* towards the orthogonal motion directions at -90° and 90°, which a criterion shift in an SDT model would not be able to explain. As a result, even if an SDT model could capture our data to some extent, it would not be expected to generalize to other settings in which this exact decision axis is not used – for example a reproduction task, or a task dividing the response region differently, even with identical stimuli – and it would be difficult to reconcile with previous literature showing a continuous perceptual biasing effect of the slow-motion prior.

3. It seems that even the best-performing WPPCE model shows noticeable discrepancies from the empirical data (as visible in Figures 7C & 7D). These remaining gaps might suggest that either important computational principles are still missing from the models or that the experimental paradigm introduces complexities not captured by the models. Further model exploration or a discussion of these limitations seems to be necessary.

We agree that there is still complexity that could be added to the models to more completely capture the data, however exploring all of those possibilities is out of the scope of this work. We now discuss the limitations of the modelling in the Discussion (750-757).

We also realized that the original figure format in Figures 7C-D (and all similar figures) was misleading in terms of the quality of the model fits. In the original figures, some of the squares of the grids reflected only a couple of trials (just two trials in the most extreme case), making them problematic to strongly interpret. We have now changed the structure of these figures, in part to improve the ease of comparison between models and data (in response to the reviewer suggestion below), but also to capture the number of trials that fall at each data point. The new figures reveal that for the data points with more trials, the WPPCE model fits much better than in sparsely sampled regions.

Minor comments:

1. Did the authors examine the relationship between the strength of the prior and the strength of confidence bias across participants? It might also be interesting to see whether individual difference exists.

We thank the reviewer for this interesting suggestion. To investigate this, we tested for a correlation across participants between the degree of criterion shift in the Bias condition from the (naive) psychometric analysis (with individual fits shown in Fig. S2) and the degree of confirmatory confidence bias observed (as measured by the α parameter of the WPPCE model, fit per participant). Large values of mu indicate a strong effect of the prior (perceptual bias) and large values of α indicate more use of the PCE rather than the posterior evidence, and hence a stronger confidence bias (relative to first-order decisions). We ran a Spearman's rank correlation and found a trend towards a moderate positive correlation between the variables, $\rho(24) = 0.40$, $p=0.055$, however this did not reach significance. This may tentatively indicate that there is a relationship between the strength of long-term priors and the degree of confirmatory use of prior-congruent evidence in confidence, but we cannot reasonably draw that conclusion from these data. While out of the scope of this work, we believe this would be interesting to investigate further in a larger sample that is more appropriate for an individual differences approach. We now mention it in lines 557-561 and include the analysis and a new figure in Appendix S4.

2. The paper would benefit from a more explicit discussion of how the findings relate to the broader Bayesian confidence hypothesis and Bayesian framework (Bowers & Davis, 2012; Griffiths et al., 2012).

We agree that this was lacking and have added a paragraph to the Discussion, contextualizing our approach in the broader literature on Bayesian confidence (701-717). It was not our primary focus to evaluate the Bayesian confidence hypothesis in totality, but rather to use the Bayesian confidence model as a tool to explore the influence of long-term priors in perceptual decisions and confidence. So, in line with discussion in Griffiths et al., 2012, we take the approach of using the Bayesian confidence model as a benchmark against which to compare confidence behaviour, without arguing that those are truly the computations being executed by the brain. Further, we really

focus on using it as a benchmark to investigate the role of the slow-motion prior across different processing levels, with the perceptual decision level as our comparative baseline.

3. The confidence models have different numbers of parameters, so AIC alone may not provide a complete picture. BIC results would offer complementary information for model comparison. At least consider adding it in the supplementary.

We have included BIC results in Appendix S7. This did not change any of the conclusions but we agree that it now provides a more complete picture.

4. The experimental data heatmap only appears in Figures 4A and 7D, making it difficult to compare model predictions to the data. Maybe a difference maps between experimental data and model predictions when presenting results from different models would help?

We have now changed the model results figures (now Fig. 3D, 4B, 5B, 6B-C, 7B) such that it is possible to compare model predictions directly to the data. The new visualizations also allow for a clearer depiction of how many trials fall in each grouping of stimulus settings/responses, for a more complete depiction of the model results.

5. A complementary way to examine the relationship between decision bias and confidence is through mixed-effects regression. One could regress confidence on false alarm rate (as a proxy for bias), accuracy, and RT. There is preliminary data suggesting that while bias positively correlates with confidence when considered alone, it negatively predicts confidence when accuracy is controlled, indicating that participants may metacognitively discount bias. Including such an analysis or discussing it would enhance the mechanistic understanding of the observed bias.

We agree that this can be a fruitful complementary approach, but data from the confidence forced-choice paradigm are not very well-suited to this type of analysis. That is because, instead of having a confidence judgement per trial that depends on the stimulus and response accuracy (and maybe RT and bias), we have a confidence choice that depends on the comparison of *two* stimuli with response accuracies and RTs and biases, which makes a regression model very complex. However, the different modelling approaches that we use in the original manuscript (non-parametric confidence analysis, cfc-model, as well as the Bayesian-based models) do already account for accuracy when assessing the influence of the perceptual bias. So, our results are not in line with a metacognitive discounting of that bias, since confidence choices *favor* the condition with strong perceptual bias after accuracy is controlled. However, this pattern of results may be related to the fact that our participants were not aware of their perceptual bias (which was confirmed in the debriefing). It is possible that if participants were made aware of their bias, they may be able to use their metacognitive system to correct for it. Another possibility is that participants do not metacognitively discount a bias of this type – namely, stemming from a prior reflecting natural statistics – because in most cases this prior should actually *help* constrain our interpretations. This may not be the same for different types of cognitive bias, which might be more adaptive to discount at the metacognitive level. We now discuss these possibilities in the Discussion (676-684).
