# Supplementary material for: Long-term perceptual priors drive confidence bias that favors prior-congruent evidence
Source: PLoS Comput Biol. 2025 Dec 22;21(12):e1013826. doi: 10.1371/journal.pcbi.1013826 (PMC12753060; doi:10.1371/journal.pcbi.1013826)
Supplement: Attachment — Submitted filename: Response_to_Reviewers_auresp_2.pdf [file pcbi.1013826.s003.pdf]

Dear Dr. Einhäuser,

Please find enclosed a second revised version of our manuscript “Long-term perceptual priors drive confidence bias that favors prior-congruent evidence”, by Marika Constant, Elisa Filevich, and Pascal Mamassian. We thank you for the opportunity to submit this revised version.

In response to the previous version, reviewers made several minor suggestions that we have addressed in our responses below, and in changes to the manuscript. The key changes include:

- Simplifying the previous 4-panel figures showing the confidence data and model results such that they are collapsed into a single panel, for ease of interpretation
- Including a more explicit discussion of our choice to test a posterior sampling model as our ‘Dist-To-Crit’ model, and including a new posterior mean model in Supporting Information
- Clarifying the implementation of the Decoupled Prior model

We copy below the reviewers’ comments and our responses (in blue). We would like to thank the reviewers again for their comments, which we feel have helped us improve the manuscript. We would also like to thank you again for considering our manuscript for publication.

Sincerely,
Marika Constant, Elisa Filevich, and Pascal Mamassian

Reviewer #1: Re-review of "Long-term perceptual priors drive confidence bias that favors prior-congruent evidence"
Reviewer: Michael Landy

This revision is much-improved with additional models and clarification and is pretty much ready for publication. I still have my reservations about the prior model (in particular about "confidence boost") and I still find the winning model a bit of a hack, averaging two models, one of which overpredicts and the other underpredicts. But, all in all, it's a clever project and quite interesting and thorough.

Thank you for the positive feedback about the revised manuscript.

Very minor comments (numbering from the no-track-changes version):

Line 40: "assumed to have Gaussian noise" is a phrase that's applied to the likelihood and to the prior. I don't think of the width of the prior as "noise", since the prior is a fixed quantity (from the decision-maker's perspective), and thus not noisy. It's width is a component of knowledge of world statistics, but when used, it's just fixed.

We agree and have clarified the wording (line 41).

428: The brain presumably DOES need to know the full distributional properties in order to carry out sampling, so this seems not quite true. I still don't love the sampling idea as a process model, don't know of prior papers that support posterior sampling for this and, personally, would prefer to see a posterior mean or mode model included or at least discussed.

We understand the skepticism about posterior sampling, which we agree is still debated in the literature. However, we chose to include it since it is both computationally simpler than the full posterior probability, posterior mean, or posterior mode (which require either integration or global search), and is argued to be biologically plausible (e.g. Orbán et al., 2016).

We now address this point in the revised manuscript (lines 438-441; 451-454), copied below, and also include a posterior mean model in Supporting Information (Appendix S5) for completeness, which yields similar results.

"However, another possibility for computing confidence is to base it off of a single sample from the posterior as a heuristic, in which case the brain does not need to perform the full integration (see, e.g., (36)). Such posterior sampling is still debated in the literature, but there is some evidence in support of it (37)."

"Beyond posterior sampling, other possible heuristics include basing confidence on the mean of the posterior distribution, but this is more computationally costly, and does not substantially change the results (Appendix S5)."

Orbán G, Berkes P, Fiser J, Lengyel M. Neural Variability and Sampling-Based Probabilistic Representations in the Visual Cortex. Neuron. 2016 Oct 19;92(2):530–43.

459-461: You say you allow for a different prior width for estimation compared to for confidence, but then the model scales the likelihood rather than the prior. Sure, this is pretty much equivalent, but reads funny.

We agree that this was confusing. We have adjusted the wording (lines 471-474, copied below).

"It is possible that the confidence data can be better explained by using a weaker likelihood relative to the prior for computing confidence than for perceptual decisions, leading to a stronger influence of the prior in confidence, similar to the pattern found in previous work (18)."

585: I'm happy about the addition of the efficient-coding model. Why do you need transduction noise? It's kind of a confidence-noise parameter, which the other models don't require. Why can't it account for confidence bias when the model predictions look pretty good?

The transduction noise in the EC model acts as noise in the mapping between the external stimulus and the internal signal. This influences primarily the first-order perceptual decision and, consequently, confidence judgments. Without this noise, the mean of the likelihood would always be exactly in line with the stimulus. This would also result in the model predicting the identical choice and confidence value on every trial with the same stimulus, which cannot account for the behavioural data. The other models all involve a very similar noise in the mapping between the stimulus and internal signal (or the mean of the likelihood distribution), which is equal to $sigma_{Likelihood}$. We have clarified this line to avoid implying that this is specifically acting as confidence noise (lines 597-600, copied below).

"Transduction noise is necessary to account for variance in perceptual decisions and confidence values across trials, as otherwise the model would predict identical outcomes every time the same external stimuli are repeated."

We have also adjusted the wording to state that the EC model *underpredicts* the confidence bias, rather than that it cannot account for it at all (line 603).

Four-panel confidence plots (several figures): Please make the y-axis scales and tick mark labels the same for all such figures.

We have simplified the confidence plots in accordance with a comment from Reviewer 2, so they are now a single panel, but we have ensured the y-axis scales and labels are the same for all such figures.

–

Reviewer #2: The authors did a great job addressing my concerns, particularly through the addition of the Efficient Coding model.

We thank the reviewer for the positive feedback on the revisions.

I have some remaining minor suggestions for the revised manuscript:

The new figure panel style for the model predictions (e.g., figure 3D and 4B etc) provide valuable information, but I found them somewhat difficult to parse. If possible, simplifying these figures or spreading the information across additional panels might improve readability. Maybe removing the gray background will help too. It's also possible that one could flip one of the Colors to combine the data (i.e., turn every Blue into an Orange by changing the sign on stimuli and answer), so that some panels and lines can be eliminated but I am not entirely certain that this is balanced.

We have now simplified the figures by grouping the data based on stimulus difficulty and perceptual decision accuracy, such that 'Strongly correct' trials consist of either trials with an extreme blue stimulus (targeting a 0.85 'Blue' perceptual decision probability) and a 'Blue' decision, or trials with an extreme orange stimulus (targeting a 0.15 'Blue' perceptual decision probability) and an 'Orange' decision. 'Strongly incorrect' trials consist of trials with those extreme stimulus settings but opposing perceptual decisions, and the 'Weakly correct'/'Weakly incorrect' follow the same patterns but for the less extreme stimulus settings (targeting a 0.65 or 0.35 'Blue' perceptual decision probability). This way, the figures are reduced to a single panel. Figure 3D is copied below as an example of this, including the new caption.

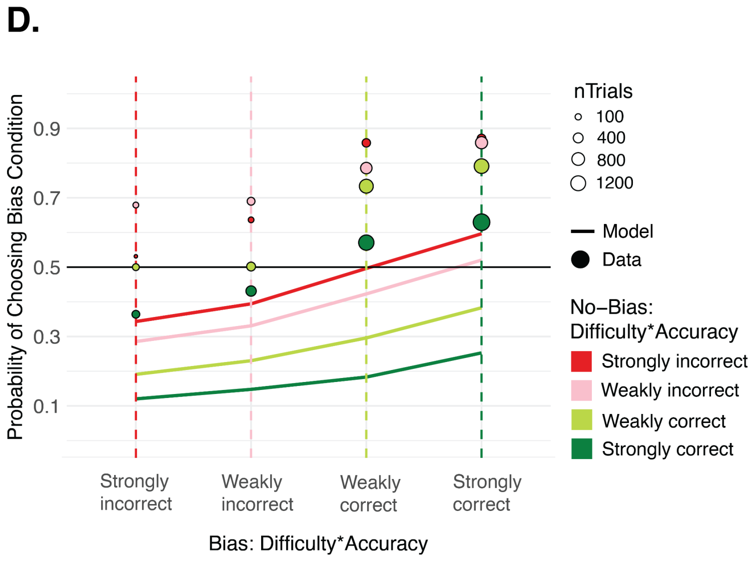

__D. Modeled confidence choice results.__ In two consecutive intervals, a Bias and a No-Bias stimulus were presented (or in the reverse order). Because confidence refers to an estimate that a perceptual decision is correct, we consider each possible combination of difficulty and perceptual decision accuracy. 'Strongly correct' refers to trials with either an extreme blue stimulus (Expected P('Blue') of 0.85) and a 'Blue' perceptual decision, or an extreme orange stimulus (Expected P('Blue') of 0.15) and an 'Orange' perceptual decision. 'Strongly incorrect' refers to trials with those extreme stimulus settings but opposing perceptual decisions. The 'Weakly correct' and 'Weakly incorrect' categories follow the same patterns but with the more difficult stimuli: Expected P('Blue') of 0.35 or 0.65. Results split further by orange versus blue stimulus settings and perceptual decisions are shown in Appendix S4. The x-axis captures this combination of difficulty and perceptual decision accuracy in the Bias condition interval —also indicated by thin, colored, dashed vertical lines—. The combination of difficulty and perceptual decision accuracy in the No-Bias condition interval is captured by color. The colors correspond to the vertical lines of the Bias condition, such that where a colored point intersects a dashed vertical line of the same color, there is the same degree of difficulty and perceptual decision accuracy across the two conditions, and an unbiased confidence observer would choose both conditions at equal rates for their confidence. The y-axis captures the confidence choice rates favoring the Bias condition. Simulated confidence choice rates from the Bayesian model are shown by the solid lines and the observed confidence data are shown by circles whose size reflects the number of trials. The overall shift upward of the confidence data points compared to the model predictions indicates that observed confidence choices favored the Bias condition more often than would be expected of the Bayesian model, which actually predicts a bias in the opposite direction.

We have still included the full 4-panel figures in Appendix S4, for completeness.

Please clarify in the methods section whether the models were fitted exclusively to the perceptual choice data, exclusively to the confidence judgment data, or jointly to both.

We have clarified this (lines 978-985, copied below).

"For the remaining modeling, we wanted to assess whether the information used in confidence computations deviates from that used in perceptual decisions, and in particular whether the use of the slow-motion prior deviates. Hence, we took the approach of fitting a model to the first-order perceptual decision data, and then exploring whether deviations from that model are needed to explain confidence. So, the first-order parameters were fit exclusively to the perceptual decisions, and any further confidence parameters (w in the Decoupled Prior model and α in the WPPCE model) were fit exclusively to the confidence choice rates (per set of perceptual responses)."

It seems to me that another possible interpretation of the WPPCE model is that instead of an "overweighted" prior, the observers are working with a less precise likelihood (i.e., the sensory evidence has degraded in working memory). Since posteriors are normalized, both overweighting the prior and underweighting the likelihood have the same effect on the subjective probability of being correct. I would appreciate it if the authors could briefly comment on this.

In the winning WPPCE model, the prior is not overweighted, but rather there is a cue proportional to the prior-congruent evidence that influences confidence, on top of the posterior. While this still involves an ‘extra’ use of the prior, it is not computationally equivalent to overweighting the prior, which is instead what is done in the Decoupled Prior Model. The differences in the effect on confidence when overweighting the prior versus when using the prior-congruent evidence cue can be seen by comparing the model predictions across these two models.

In the Decoupled Prior Model, however, we completely agree that the “overweighted” prior is equivalent to a degraded likelihood. We even implement it as a less precise likelihood rather than a more precise prior, and we agree that these two cannot be easily disentangled. We have clarified the wording (lines 471-474), also in line with a comment from Reviewer 1, to make this more transparent.

–

Reviewer #3: Thank the authors for addressing my comments. The paper looks all good now. Just one remaining thought: the authors mentioned in the reply that in their task there is no explicit sensory criterion because the reference moved from trial to trial, but my impression is that even though the reference varied, each trial still effectively defined a decision boundary. For example, the confidence could, in principle, depend on the distance between the perceived motion direction and this trial-specific criterion. I understand that the author might want to maintain a Bayesian generative framing, but it would be great to see what the authors think about it.

We thank the reviewer for the encouraging words on the revised manuscript, and for the follow-up discussion regarding an SDT approach.

We agree completely that each trial may still effectively have a decision boundary. However, what seems difficult to reconcile with an SDT framework is how that decision boundary would *shift* to account for the influence of the prior on each trial, as the influence of the prior on perception of these stimuli is constantly changing and is unpredictable before motion begins. Participants would not be able to learn or predict a bias effect such that they could viably shift their decision boundary in accordance with the slow-motion prior’s effect trial-by-trial. Further, even if we chose to model the influence of the prior as a criterion shift, this would fail to capture the particular pattern of perception in the No-Bias condition, as the unbiased criterion would not capture the expected perceptual bias towards -90° and 90°. So, though we agree one could still map this into an SDT model, we chose to model the influence of the prior in this Bayesian framing, to align better with continuous perceptual predictions, and to more intuitively capture how participants might perform the perceptual decision task.

However, confidence could depend on the distance between the perceived motion direction and a trial-specific criterion, as the reviewer mentions, but with the influence of the slow-motion prior being implemented in the perceived motion direction rather than in a criterion shift. This is what is captured in the Distance-to-Criterion confidence model.

Also, some formulas are not properly displayed (line 300 – 308).

Thank you for pointing this out. We believe this is a conversion error when automatically creating the pdf on submission, but we will ensure this is fixed in the final .docx file.
